# Spatio-temporal evolution patterns of influenza incidence and its nonlinear spatial correlation with environmental pollutants in China

**DOI:** 10.1186/s12889-023-16646-z

**Published:** 2023-09-01

**Authors:** Hao Li, Miao Ge, Congxia Wang

**Affiliations:** 1 Institute of Healthy Geography, School of Geography and Tourism, Shaanxi Normal University, Xi’an, 710119 China; 2 Department of Cardiology, The Second Affiliated Hospital of Medical College, Xi’an Jiaotong University, Xi’an, 710004 China

**Keywords:** Influenza, Spatio-temporal evolution pattern, Pollutants, Generalized additive model, Space-time cube model

## Abstract

**Background:**

Currently, the influenza epidemic in China is at a high level and mixed with other respiratory diseases. Current studies focus on regional influenza and the impact of environmental pollutants on time series, and lack of overall studies on the national influenza epidemic and the nonlinear correlation between environmental pollutants and influenza. The unclear spatial and temporal evolution patterns of influenza as well as the unclear correlation effect between environmental pollutants and influenza epidemic have greatly hindered the prevention and treatment of influenza epidemic by relevant departments, resulting in unnecessary economic and human losses.

**Method:**

This study used Chinese influenza incidence data for 2007–2017 released by the China CDC and air pollutant site monitoring data. Seasonal as well as inter monthly differences in influenza incidence across 31 provinces of China have been clarified through time series. Space-Time Cube model (STC) was used to investigate the spatio-temporal evolution of influenza incidence in 315 Chinese cities during 2007–2017. Then, based on the spatial heterogeneity of influenza incidence in China, Generalized additive model (GAM) was used to identify the correlation effect of environmental pollutants (PM_2.5_, PM_10_, CO, SO_2_, NO_2_, O_3_) and influenza incidence.

**Result:**

The influenza incidence in China had obvious seasonal changes, with frequent outbreaks in winter and spring. The influenza incidence decreased significantly after March, with only sporadic outbreaks occurring in some areas. In the past 11 years, the influenza epidemic had gradually worsened, and the clustering of influenza had gradually expanded, which had become a serious public health problem. The correlation between environmental pollutants and influenza incidence was nonlinear. Generally, PM_2.5_, CO and NO_2_ were positively correlated at high concentrations, while PM_10_ and SO_2_ were negatively correlated. O_3_ was not strongly correlated with the influenza incidence.

**Conclusion:**

The study found that the influenza epidemic in China was in a rapidly rising stage, and several regions had a multi-year outbreak trend and the hot spots continue to expand outward. The association between environmental pollutants and influenza incidence was nonlinear and spatially heterogeneous. Relevant departments should improve the monitoring of influenza epidemic, optimize the allocation of resources, reduce environmental pollution, and strengthen vaccination to effectively prevent the aggravation and spread of influenza epidemic in the high incidence season and areas.

## Background

Influenza is an acute respiratory infectious disease caused by the influenza virus with obvious seasonal characteristics. The entire world’s population is essentially susceptible to influenza, which can be transmitted by aerosol, droplets, and physical contact. The burden of disease caused by influenza is severe. According to the World Health Organization (WHO), influenza kills about 650,000 people worldwide each year. Since the 20th century, there have been four influenza pandemics, but each has had significant differences with respect to virus subtypes, disease populations, spatial and temporal distribution, and case fatality. Influenza and its complications are a serious public health problem worldwide [1, 2]. China has a higher incidence of influenza than other countries. Owing to the diversity of population, economy, environment, and other factors in different regions of China, the current unclear space-time evolution law of the influenza epidemic and complex pathogenic factors have brought great obstacles to the prevention and control of the influenza epidemic in China. In recent years, infectious respiratory diseases have become more serious. Respiratory diseases such as coronavirus disease 2019 (COVID-19), which are still doing the rounds, are mixed with influenza. The early symptoms for both are very similar. During the COVID-19 pandemic, an increase in the number of cases of influenza with similar symptoms likely significantly increased the control of respiratory infectious diseases. Research on the spatio-temporal evolution of influenza and environmental risks may significantly reduce the disease and economic burden in China.

Previous studies had shown that influenza in China had obvious seasonality, with a significant increase in the number of influenza infections in winter and spring. Studies conducted in different regions of the world have found regional variations in influenza seasonality. In tropical and subtropical regions, there were many peaks in influenza incidence [3]. In the middle latitudes that experience distinct seasons, influenza was more prevalent in winter and varied with latitude [4]. China has a large area and diverse weather patterns, and the influenza incidence in China also showed regional differences. A number of studies have been conducted from the aspect of epidemiological characteristics [5], regional clustering [6, 7], and prediction [8] of influenza. Few studies had combined time series and spatial heterogeneity to further investigate the spatio-temporal evolution pattern of influenza incidence across the country.

Environmental epidemiological studies have shown that exposure to environmental pollutants significantly affects the risk of influenza [9, 10]. With the development of spatio-temporal statistical analysis techniques, many mathematical models have been used to study the environmental risk factors of influenza on a macro scale and their effects. Su et al. used wavelet coherence analysis and GAM to explore the short-term and delayed effects of environmental pollutants based on daily influenza incidence and pollutant data in Jinan, China [11]. Liao et al. analyzed the excess risk and lag effect of various environmental pollutants on influenza incidence by using a GeoDetector model and distributed lag nonlinear model [12]. Zhang et al. analyzed the community-level association between PM_2.5_ and influenza using random forest and spatio-temporal Bayesian model [13]. Most studies focused on the time series of influenza incidence to study the effects of short-term or long-term exposure to environmental pollutants. The exploration of spatial heterogeneity of environmental pollutants and influenza incidence in different regions is still in its early stage. One study showed that the influence of environmental pollutants on influenza had obvious regional differences [14]. At present, GWR and several spatial models are the main research methods for the spatial correlation effect between environmental factors and influenza incidence. GWR was based on linear models without considering the nonlinear effects of influencing factors. The GWR model showed poor adaptability to the multicollinearity of environmental factors with complex interactions. GAM is usually used to study the short-term lag effect of environmental factors on disease. Given the characteristics of its additive model, it can also better explore the nonlinear spatial correlation effect of environmental factors and influenza incidence [15].

In this study, an STC model was established based on the time series and spatial heterogeneity of influenza incidence, to explore the spatio-temporal evolution of influenza incidence in China. Combined with GAM, the nonlinear spatial correlation effect between environmental pollutants and influenza incidence was explored. This study will help to better understand the developmental trend of the influenza epidemic in China, clarify the impact of environmental pollutants on influenza, and provide a basis for the prevention and control of the influenza epidemic.

## Materials and methods

### Influenza incidence data

Influenza incidence data in China from 2007 to 2017 were obtained from The Data Center of China Public Health Science of Chinese Center for Disease Control and Prevention (CDC http://www.phsciencedata.cn/Share/index.html?ed9df15d-9e67-49c4-9416-1f179d198118). The database contains influenza incidence data from 2007 to 2017 from 315 municipalities in 31 provinces of China. Some data of a small number of municipal regions are missing. Due to difficulties in data acquisition, data of Hong Kong, Macao and Taiwan are not included.

### Environmental pollutants data

The concentration data of environmental pollutants came from 1,496 national air quality monitoring stations, including the longitude and latitude of the stations as well as the daily concentrations of PM_10_, PM_2.5_, SO_2_, NO_2_, CO and O_3_ in 2017. However, some time period data were missing. In this study, Python was used to carry out data cleaning, and some sites with special values and seriously missing data were eliminated, and then sliding average processing method was used to calculate the annual average concentration of six air pollutants from national ambient air quality monitoring stations in 2017. The Kriging method was used to interpolate the site data to cover the entire study area, followed by sectional statistics to match the influenza incidence data. Table 1 shows the summary of environmental pollutants used in this study.

**Table 1:** Summary of environmental pollutants

	Mean	SD	Minimum	P25	Median	P75	Maximum
CO (µg/m^3^)	1.078	0.283	0.552	0.865	1.015	1.238	2.175
NO_2_ (µg/m^3^)	28.722	7.863	13.114	22.675	28.039	33.520	47.564
O_3_ (µg/m^3^)	97.740	10.230	75.567	89.353	96.226	104.847	122.909
PM_10_ (µg/m^3^)	86.254	32.701	33.460	61.450	83.799	101.976	267.537
PM_2.5_ (µg/m^3^)	48.999	16.322	16.443	37.145	47.692	56.697	96.901
SO_2_ (µg/m^3^)	24.915	11.887	4.313	16.976	21.839	29.798	62.730

### Space-time cube model (STC)

**Fig. 1:**
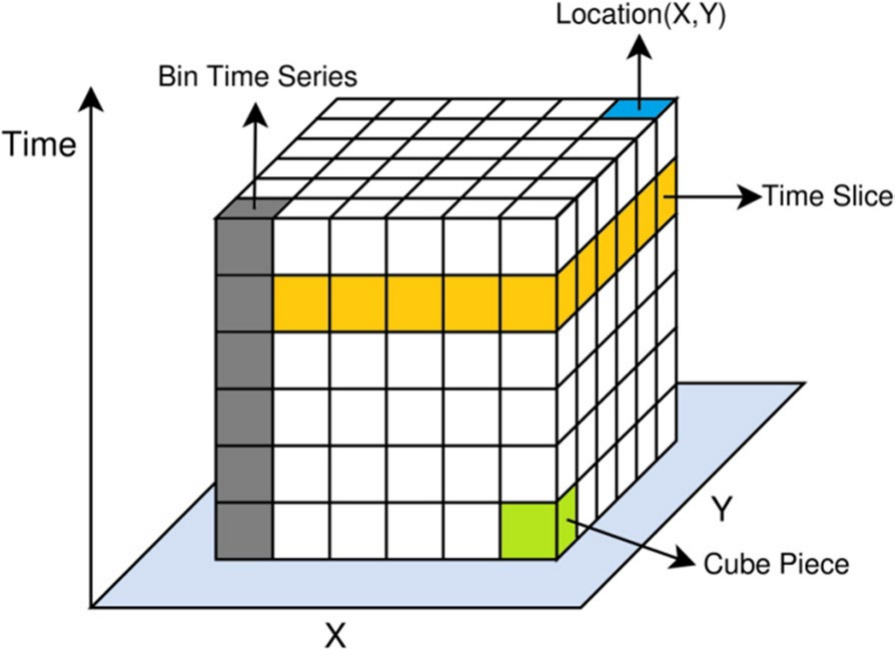
Space-time cube model

Figure 1 shows the Space-time cube model. The STC was proposed by Hagerstrand in 1970, and further discussed by Rucker and Szego. The STC can show how influenza incidence changes over time in geographic space, and identify the spatio-temporal evolution pattern of influenza incidence through spatio-temporal clustering and hotspot analysis. On the basis of two-dimensional plane space, a one-dimensional time axis was added to the STC, and time steps were used to divide the STC into multiple time slices, and then a three-dimensional cube combining the spatial location and time series was formed. It can represent changes of influenza incidence at a given location over time steps.

Based on the established STC, Getis-Ord Gi* was used to analyze the spatial hot spots of each time slice, and then Mann-Kendall statistical method was used to measure the trend of the bin time series in each location [16], so as to identify the spatio-temporal aggregation pattern. The Mann-Kendall trend test is as follows:

For a bin time series with a sample size of n, each cube piece value on the bin time series was calculated and compared with the previous cube piece value, and the value was assigned according to its comparison results.

$$diff=\left\{\begin{array}{c}1, {x}_{j}>{x}_{i}\\ 0, {x}_{j}={x}_{i}\\ -1, {x}_{j}<{x}_{i}\end{array}\right.$$

Where *diff* is the assignment, *i* is a cube piece, *j* is the next cube piece.

The results of each pair of time periods were summed:

$$SUM=\sum_{i-1}^{n-1}\sum_{j=i+1}^ndiff$$

The expected result of *SUM* is 0, indicating that there is no trend in influenza incidence at this location over time. Based on the variance of the values in the STC bin time series, the observed *SUM* was compared with 0 to determine whether the difference was statistically significant. The trend of each bin time series will be recorded as *Z*-score and *p*-value. By categorizing *Z*-scores and *p*-values, The spatio-temporal evolution pattern of influenza incidence can be classified as New, Consecutive, Sporadic, Oscillating, Intensifying, Persistent, Diminishing, Historical hot or cold spots, as well as one pattern with no significant hot or cold spots characteristics [17]. Table 2 shows the classification definitions of each hot and cold spot pattern.

**Table 2:** Definitions of the Hot/Cold spot patterns

Pattern name	Definition	Pattern name	Definition
New Hot/Cold Spot	A location that is a statistically significant hot/cold spot for the final time step and has never been a statistically significant cold/hot spot before.	Intensifying Hot/Cold Spot	A location that has been a statistically significant hot/cold spot for 90% of the time-step intervals, including the final time step. In addition, the intensity of clustering of high/low counts in each time step is increasing overall and that increase is statistically significant.
Consecutive Hot/Cold Spot	A location with a single uninterrupted run of at least two statistically significant hot/cold spot bins in the final time-step intervals. The location has never been a statistically significant hot/cold spot prior to the final hot/cold spot run and less than 90% of all bins are statistically significant hot/cold spots.	Persistent Hot/Cold Spot	A location that has been a statistically significant hot/cold spot for 90% of the time-step intervals with no discernible trend in the intensity of clustering over time.
Sporadic Hot/Cold Spot	A statistically significant hot/cold spot for the final time-step interval with a history of also being an on-again and off-again hot/cold spot. Less than 90% of the time-step intervals have been statistically significant hot/cold spots and none of the time-step intervals have been statistically significant cold/hot spots.	Diminishing Hot/Cold Spot	A location that has been a statistically significant hot/cold spot for 90% of the time-step intervals, including the final time step. In addition, the intensity of clustering/low counts clustering in each time step is decreasing overall and that decrease is statistically significant.
Oscillating Hot/Cold Spot	A statistically significant hot/cold spot for the final time-step interval that has a history of also being a statistically significant cold/hot spot during a prior time step. Less than 90% of the time-step intervals have been statistically significant hot/cold spots.	Historical Hot/Cold Spot	The most recent time period is not hot/cold, but at least 90% of the time-step intervals have been statistically significant hot/cold spots.
No Pattern Detected	Does not fall into any of the hot or cold spot patterns defined.		

Based on the established STC, the time series clustering method used the k-means clustering algorithm to classify the spatial units with similar time series in the STC, so as to identify the temporal variation characteristics of influenza incidence in different cities. In this study, the cluster number was set as 5 categories.

### Generalized additive model (GAM)

Poisson regression generalized linear model had been widely used in the study of the harm of environmental pollution to human health, which can quantitatively evaluate the intensity of the correlation effect between environmental pollutants and health. However, the generalized linear model assumes that the observations are independent. Health data on time series are all autocorrelation, so in the 1990s, Hastie and Tibshirani extended the generalized linear model to the generalized additive model. This model was more flexible and can estimate the relative risk, so it had been widely used in recent years to analyze the harm of environmental pollutants to human health. Influenza incidence usually follows Poisson distribution, so Poisson regression was selected as the link function, and GAM was established with the influenza incidence of each city in China as the dependent variable and each environmental pollutant as the independent variable. The model used is shown as follows:

$$Log\left[E\right(Y\left)\right]=s({PM}_{2.5}, df)+s({PM}_{10}, df)+s(CO, df)+s({SO}_{2}, df)+s({NO}_{2}, df)+s({O}_{3}, df)+Intercept$$

Where *E(Y)* represents the expected incidence of influenza; *s()* stands for smoothing function; *df* is the degree of freedom of the function.

The minimum AIC value was used to select the optimal GAM. The establishment of the model, the calculation of relative risk and the plotting were completed by R software 4.1.1 (mgcv, gamRR, nlme package).

## Results

### Time series of influenza incidence in China

**Fig. 2:**
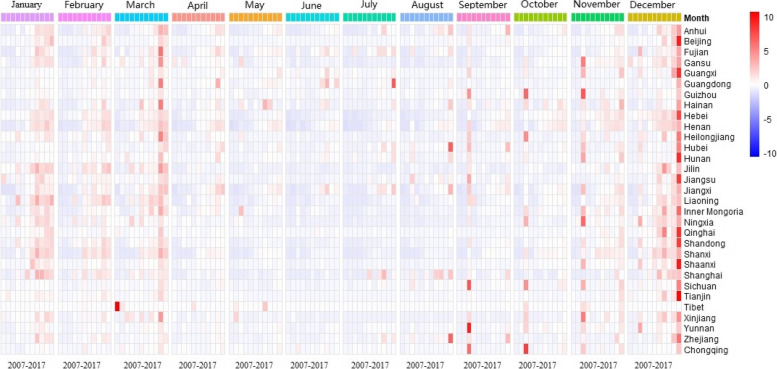
Time series of influenza incidence in 31 provinces of China from 2007 to 2017

Figure 2 shows the time series of influenza incidence by province in China from 2007 to 2017. We normalized the influenza incidence by province. According to the statistics of time series, influenza incidence in most areas of China had obvious seasonal changes, and influenza occurred frequently in winter and spring. The 2009 influenza pandemic lasted from September to January of the following year. In recent years, many cases of influenza started from November to March of the next year, and the incidence was significantly higher than other months in summer and autumn. After March, the influenza incidence decreased significantly, and only sporadic outbreaks occurred in some areas. From a year-on-year perspective, in the past 5 years, the influenza incidence in various regions of China had increased rapidly. In December 2017, China experienced a relatively severe influenza epidemic. Influenza seasonality was not obvious in some areas. For example, Shanghai saw a peak of influenza incidence in July and August, Jiangxi Province showed no significant difference in monthly influenza incidence, and Guangdong Province had a high influenza incidence in June and July.

### Spatial and temporal patterns of influenza incidence at municipal level in China

Based on the fixed locations of 315 cities in 31 provinces of China from 2007 to 2017, the STC of influenza incidence was established, and the blank cube piece was filled based on the spatio-temporal adjacent elements. Finally, 315 bin time series and 11 time slices were obtained. According to the Mann–Kendall trend analysis, the average *Z*-score was 2.316, and the *p*-value was 0.008. It can be concluded that the average influenza incidence in China showed an increasing trend over time. We realized the visualization of the STC through the emerging hot spots analysis and time series clustering.

**Fig. 3:**
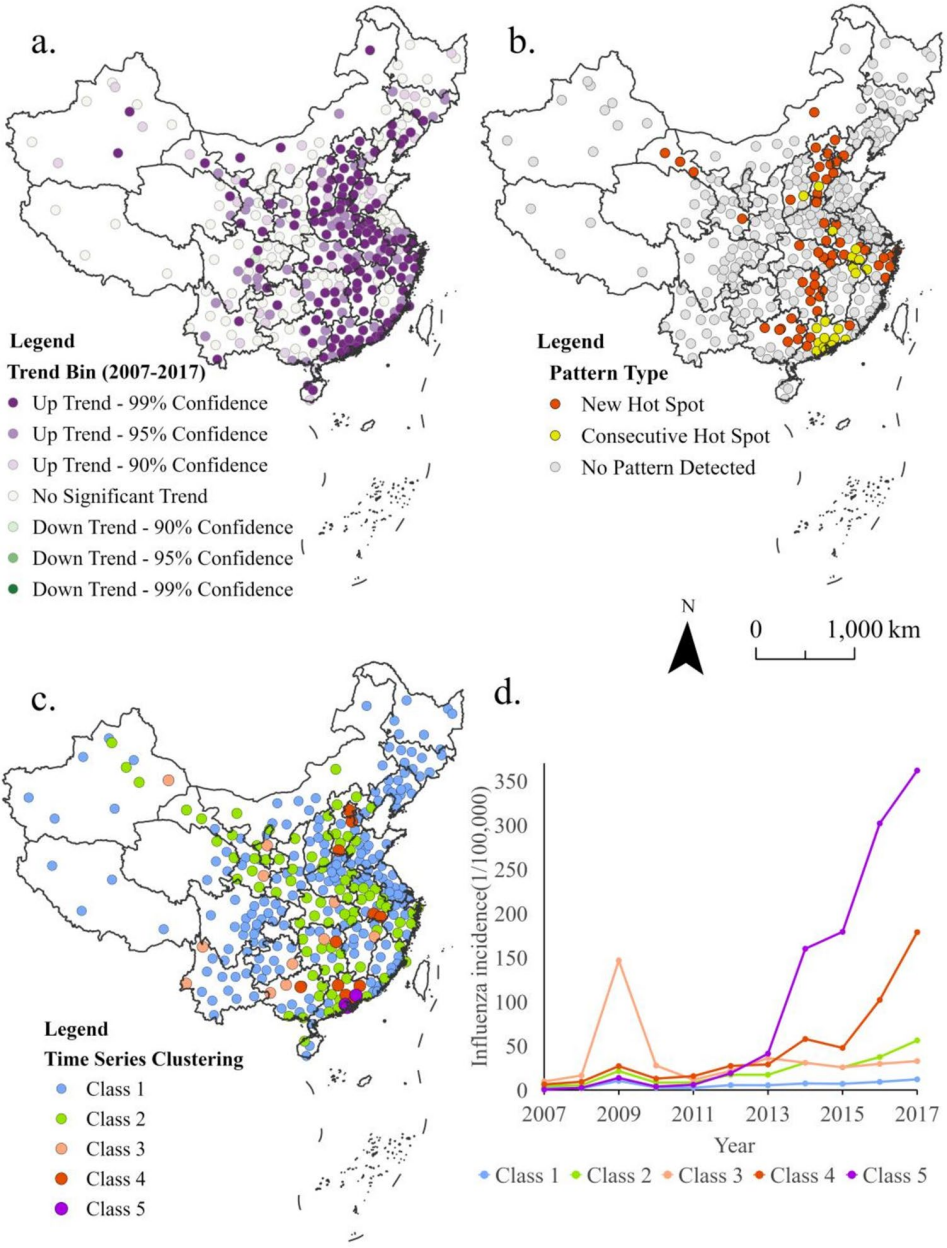
Spatial and temporal patterns of influenza incidence in China

Figure 3 shows the spatio-temporal pattern evolution of influenza incidence in China based on STC. The influenza incidence in China from 2007 to 2017 showed an increasing trend in 221 cities, accounting for 70% of the cities in China (Fig. 3a). Only Deyang in Sichuan Province showed a downward trend. The increasing influenza incidence was more obvious in the coastal areas of south China and central China. The influenza incidence fluctuated in most areas of northwest and southwest China, and some areas showed an upward trend. This indicated that the disease burden of the influenza epidemic had gradually increased in the past 11 years, and the concentrated areas of influenza epidemic have gradually expanded, which has become a serious public health problem. We explored the spatio-temporal evolution pattern of national influenza incidence through emerging hot spots analysis.

Figure 3b shows that the hot spots for high influenza incidence gradually expanded outward from three persistent hot spots in Guangdong, southern Hebei, southern Shanxi, and southern Anhui. Influenza epidemic hot spot areas had expanded from 21 consecutive hot spots (the nearest two consecutive adjacent time steps are hot spots) to 56 new hot spots (the last time step is a hot spot and has not previously been shown as a hot spot). A number of high-value aggregation regions had formed such as Beijing-Tianjin-Hebei, South China, and eastern Central China, with a “+” banded distribution. Cold or hot spot patterns were not identified in other regions.

By time series clustering, we divided regions with similar time series influenza incidence into five groups. Figure 3c and d show the spatial distribution and time change curve of the spatial and temporal clustering category of influenza incidence in China. The curve shows that most parts of China experienced a relatively mild influenza epidemic in 2009. Class 1 mainly includes Northeast China, Southwest China, Shandong, Jiangsu, Shanxi, northern Henan, northern Jiangxi, and Fujian, which showed a low level of incidence rate or slight increase after the influenza epidemic in 2009. The regions included in Class 2 are significantly similar to the hot spots in Fig. 3b, mainly including Beijing-Tianjin-Hebei, South China, eastern Central China, Shaanxi, Gansu, and Ningxia. These regions showed a slow rise in influenza incidence in recent years. According to Fig. 3c, Class 2 is roughly distributed around high value categories, so the rise of its influenza incidence may be because of the diffusion effect of influenza hot spots. Class 3 represents a small number of regions with a serious influenza epidemic in 2009, which was distributed sporadically. In the following year, the influenza incidence in these regions dropped rapidly, and had not increased significantly in recent years. Class 4 and Class 5 represent the regions where the influenza incidence had increased significantly in different degrees, mainly in Guangdong, parts of Beijing-Tianjin-Hebei, and southern Anhui. In recent years, the influenza epidemic trend in Guangdong was the most severe. It had risen sharply since 2012, showing a rapidly increasing trend and a clustering effect. The high incidence had spread to surrounding areas. The influenza incidence in central Guangdong, southern Anhui, Beijing, and some regions were significantly different from other regions, showing a strong epidemic trend in recent years. Class 4 and Class 5 were the core of hot spots and may be the source regions of the influenza epidemic.

### The correlation effect between environmental pollutants and influenza incidence

Based on the annual average data of six air pollutants and the influenza incidence data in 315 cities in China in 2017, GAM was used to fit environmental pollutants and influenza incidence. The optimal degree of freedom of each pollutant in the model and the model statistics are shown in Table 3.

**Table 3:** GAM model statistics

Smooth Items	df
s(PM_2.5_)	8.831^***^
s(PM_10_)	8.757^***^
s(CO)	8.82^***^
s(SO_2_)	8.519^***^
s(NO_2_)	8.906^***^
s(O_3_)	8.771^***^

*R*^2^ = 0.526; Deviance explained = 60.3%; Intercept = 3.03304***; AIC = 71.329; ^***^Significance level *P* < 0.001

**Fig. 4:**
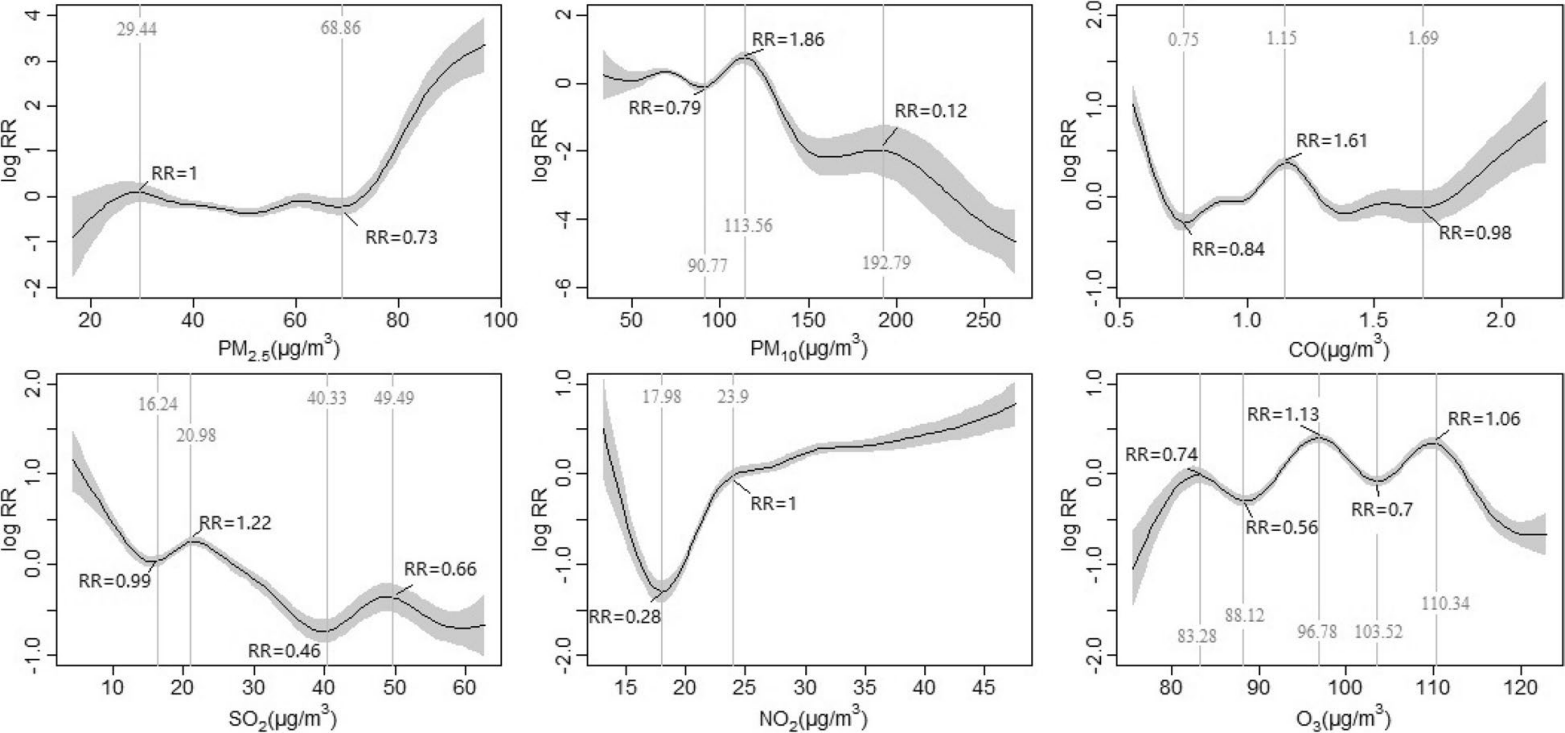
Correlation effect curve between environmental pollutants and influenza incidence in China

The gamRR package was used to determine the relative risk (RR) of the inflection point. Figure 4 shows the non-linear correlation between environmental pollutants and influenza incidence. The curve of the relationship between PM_2.5_ and influenza incidence generally showed an upward trend. A concentration of 29.44 µg/m^3^ and RR = 1 indicated that the correlation between PM_2.5_ and influenza incidence was weakened after reaching this concentration range. When the concentration reached 68.86 µg/m^3^, RR began to rise rapidly with the increase of PM_2.5_ concentration, indicating that high concentration of PM_2.5_ may have a strong influence on the increase of influenza incidence. The correlation of PM_10_ was roughly negative. RR reached its maximum at the PM_10_ concentration of 113.56 µg/m^3^, suggesting that PM_10_ would have the strongest effect on influenza onset at this concentration. Subsequently, the correlation between the two decreased with the increase of concentration and showed a negative correlation when the concentration was higher. The low concentration of CO showed a weak negative correlation with influenza incidence, and the positive correlation effect gradually became prominent with increasing concentration, and reached the regional peak when the concentration of CO was 1.15 µg/m^3^. Then, it gradually showed no association. At higher concentrations, the curve of association between CO and influenza incidence increased rapidly. SO_2_ was roughly negatively associated with influenza incidence. At low concentrations, SO_2_ likely causes a slight increase in influenza incidence, with a small excess risk. As the concentration of SO_2_ increased, the association with influenza incidence showed moderate protective effect, with the strongest protective effect at a concentration of 40.33 µg/m^3^. NO_2_ was strongly associated with influenza incidence at low concentrations, showing protective effects below 23.9 µg/m^3^ and the lowest relative risk at 17.98 µg/m^3^. The positive correlation effect of NO_2_ became stronger when the concentration exceeded 23.9 µg/m^3^. The correlation between O_3_ and influenza incidence is complex. The RR value fluctuates around 1, indicating that the correlation effect of O_3_ is not strong. It only shows weak protective effect at 88.12 µg/m^3^ and some small range concentrations.

## Discussion

With the increase of the global population, the acceleration of urbanization, the significant increase of population density and mobility, the global influenza epidemic had been increasing year by year. After the severe COVID-19 epidemic had leveled off, the level of influenza virus activity in China appeared to have a significant upward trend in early 2023. After experiencing the relatively serious COVID-19 epidemic, residents had shown significant contempt for the risk of influenza epidemic. In recent years, serious influenza epidemic had almost swept the country, with influenza epidemic occurring more frequently and on an increasingly large scale. Studies had shown that most environmental pollutants can increase the risk of influenza [18]. Clarifying the spatial and temporal evolution pattern of influenza incidence and the correlation effect with environmental pollutants will effectively promote the prevention and control of influenza epidemic and had positive significance for the protection of people’s health.

According to the statistics of time series, this study found that influenza incidence in most areas of China had obvious seasonal changes, and influenza occurred frequently in winter and spring. In recent years, many cases of influenza begin from November to March of the next year. Other studies on mainland China also found the same time series results [19, 20]. A study on influenza incidence in the United States based on weekly time series found that the influenza incidence peaked from the 48th week of each year to the 8th week of the following year, and remained relatively stable until the middle of the year. The high incidence period was slightly shorter than that in China, but the seasonality was similar [4]. The spatial and temporal patterns of influenza in similar climate regions around the world also had similar characteristics [21–23]. Influenza seasonality may vary in some areas depending on the type of virus circulating, and there may be an unobvious summer peak [24]. Low temperature in winter and spring will increase the pressure of trachea and nasopharynx. Low temperature and dry air may lead to dehydration of the human nasal mucosa and facilitate easy adhesion of bacteria and viruses, thus increasing the risk of respiratory infectious diseases [25]. This study found that the influenza incidence in China was increasing year by year, but there were significant regional differences in the increment pattern. The influenza incidence in different regions had spatiotemporal heterogeneity, which generally showed a high concentration area and a diffusion trend. Regional studies in recent years carried out more detailed identification of high-prevalence areas of influenza and explored the influencing factors for each region [26–28]. The results showed that influenza, as a respiratory infectious disease, had a very strong clustering and long-term epidemic situation [29]. Moreover, there were complex differences in the types of epidemic strains, climate characteristics, population mobility, health conditions, and residents’ living habits among regions, which may be the reason for the heterogeneity of influenza incidence on a large scale [30]. These studies on spatial patterns of influenza incidence can quickly identify key areas and epidemic sources and provide effective reference for optimal allocation of resources. In general, the influenza incidence in China not only followed seasonal development but also increased year by year since 2012. Our data revealed a very serious epidemic by 2017. According to reports in recent years, the influenza epidemic in China is still at a high level, despite the relatively strict prevention and control of COVID-19. Relevant authorities need to pay considerable attention to prevent the worsening of the influenza epidemic and take urgent measures (such as timely vaccination) to curb the epidemic situation.

Air pollution has always been a universal concern worldwide. The World Health Organization reported that air pollution exposure can cause 7 million premature deaths each year, and some diseases can even lead to severe disability, resulting in a huge burden on families and society. In the past decade, China made remarkable progress in the prevention and control of air pollution. However, even though some polluting enterprises had actively changed to cleaner models, the problems of industrial emissions and vehicle exhaust were still significant and posed a great threat to public health. Studies had shown that the correlation between air pollution and influenza incidence had become a consensus, but there was no clear research result on the influencing mechanism, and its correlation effect was still unclear [31]. Some studies reported the short-term effects of air pollution on the onset of influenza based on time series. They found that air pollution had a lag in the risk of influenza among residents. After experiencing short-term exposure to high concentrations of air pollution, the increase in the risk of illness tended to lag for a week or even longer, but almost no more than a month, and different regions exhibited different lag effects [27, 32]. Most recent studies have focused on the short- and medium-term effects of time, and there is a general lack of studies on the spatial heterogeneity of long-term exposure of environmental pollutants in different regions. Some studies indicated that there were regional differences in the long-term exposure effect of spatial environmental pollutants (one year or longer), and its spatial impact was uneven, which may be related to the inconsistency of many studies [33, 34].

Based on GAM, this study explored the nonlinear spatial correlation between environmental pollutants and influenza incidence. It was found that all environmental pollutants and influenza incidence showed a nonlinear correlation, and their relationship curves were not always in the same direction. At low concentrations, PM_2.5_ was not strongly associated with influenza incidence and may even have a slight protective effect; whereas, its positive correlation developed rapidly at high concentrations. Unlike PM_10_, PM_2.5_ can enter the alveoli and be deposited for several years, which may lead to respiratory system damage and make the human body more susceptible to influenza virus infection or aggravation of illness [35, 36]. At high concentrations, PM_10_ showed negative correlation, and the same characteristics were observed in some other studies [37, 38]. They believed that high concentration of PM_10_ may reduce UV radiation and thus reduce respiratory system damage. PM_10_ has a large particle size and cannot easily cross the human barrier, rather it can be easily eliminated by the human body. At high concentrations, it may increase enzyme activity and reduce symptoms. The influencing mechanism of PM_10_ is not clear at present, and it may also interact with other pollution factors [39]. In general, CO showed a positive correlation, but at low concentration, there may be a negative correlation, which is consistent with some studies on the pathogenesis of CO [40]. Inhalation of CO will cause damage to human respiratory epithelial cells, which is the first barrier against viruses [41]. However, low concentrations of CO may have antibacterial properties, which may be beneficial to build resistance to influenza infection [42]. The correlation of NO_2_ was similar to that of CO. Although some studies believed that NO_2_ had no correlation with influenza [43], others reported that NO_2_ had a positive effect [44]. One study estimated that about 14% of influenza cases could be attributed to excessive NO_2_ exposure (over 40 µg/m^3^) [45], which was partly consistent with the results of this study. This study found that the correlation between SO_2_ and influenza incidence was negative, which was different from other studies. However, some studies also reported this phenomenon. They believe that acidic environments are not conducive to virus survival and may reduce viral transmission [37]. Considerable research has been carried out on the influence of SO_2_ on the respiratory system, most of which believe that the effect of SO_2_ should be positive. The results of this study found that O_3_ may have no correlation with influenza incidence or only have a slightly negative correlation. Some studies believed that it may be related to the bactericidal properties of O_3_ [18], while others observed a positive correlation [46]. Many research results showed inconsistency. Epidemiological analysis suggests that the correlation between environmental pollutants and infectious diseases may not be able to determine their mutual influence and response. Furthermore, there was a time lag effect between the two, and different regions and different types of environmental factors had great differences in the effect of time. Although we had greatly removed the effects of short-term effects by taking an annual average, environmental pollutants may still cause damage by accumulating in the human body for many years. Environmental pollutants also have seasonality, and the pathogenic mechanism of influenza is complex, which is not only affected by environmental pollutants. At present, there are still large gaps in knowledge regarding environmental toxicology and its harmful effects on the human body.

Herein, we studied the influenza incidence in different time and space, determined the areas that need to be focused, and clarified the nonlinear spatial correlation effect between environmental pollutants and influenza incidence. However, our study has some limitations. First, because of the nature of data acquisition, we used the influenza incidence data of 315 cities in 11 years when establishing the STC, and did not carry out research on a smaller time scale. The year-scale data may cause some key areas with obvious seasonal differences in influenza incidence but low overall incidence to be incorrectly identified. Second, we explored the correlation effect of environmental pollutants, without considering the confounding effects of climate, population, and economic factors, and without considering the hysteresis effect. Last, we did not have data on influenza virus subtypes; therefore, the results may not be entirely accurate. In the future, we will aim to use a smaller scale and more detailed influenza data, combined with more detailed environmental-influence mechanism studies to further explore the development trend of influenza and the environmental response mechanism.

## Conclusions

Based on STC and GAM, this study explored the spatio-temporal evolution patterns of influenza incidence in China during 2007–2017, and identified the correlation effect of environmental pollutants. The study found that the influenza epidemic in China was in a rapidly rising stage, and several regions had a multi-year outbreak trend and the hot spots continue to expand outward. The association between environmental pollutants and influenza incidence was nonlinear and had spatially heterogeneous. Relevant departments should improve the monitoring of influenza epidemic, optimize the allocation of resources, reduce environmental pollution, and strengthen vaccination to effectively prevent the aggravation and spread of influenza epidemic in the high incidence season and areas.

## Data Availability

The data of this paper comes from the public database of CDC (http://www.phsciencedata.cn/Share/index.html?ed9df15d-9e67-49c4-9416-1f179d198118).

## Abbreviations

STC: Space-Time Cube model
GAM: Generalized additive model
WHO: World Health Organization
CDC: Chinese Center for Disease Control and Prevention
GWR: Geographically weighted regression
*df*: Degree of freedom
RR: Relative risk
